# RETRACTION: Clinical Significance of the Secondary Pedicle Amputation of the Repair of Distal Defects With Pedicled Axial Flap

**DOI:** 10.1111/iwj.70551

**Published:** 2025-04-18

## Abstract

**RETRACTION:** ZhangX. , DingP. , WangG. , ChenY. , YangX. , ZhaoZ. , and BiH. , “Clinical Significance of the Secondary Pedicle Amputation of the Repair of Distal Defects With Pedicled Axial Flap,” International Wound Journal 19, no. 5 (2022): 1009–1015, 10.1111/iwj.13697.34636163 PMC9284622

The above article, published online on 12 October 2021, in Wiley Online Library (http://onlinelibrary.wiley.com/), has been retracted by agreement between the journal Editor in Chief, Professor Keith Harding; and John Wiley & Sons Ltd. Following an investigation by the publisher, all parties have concluded that this article was accepted solely on the basis of a compromised peer review process. In addition, further investigation by the publisher found that no consent statement from the patient depicted in Figure 3 had been included in the article and that the operational methods were not adequately described in the methodology. In view of the clear evidence of compromised peer review, the parties agreed that the paper must be retracted. The authors did not respond to our notice regarding the retraction.

**RETRACTION:** X. Zhang , P. Ding , G. Wang , Y. Chen , X. Yang , Z. Zhao , and H. Bi , “Clinical Significance of the Secondary Pedicle Amputation of the Repair of Distal Defects With Pedicled Axial Flap,” International Wound Journal 19, no. 5 (2022): 1009–1015, 10.1111/iwj.13697.34636163 PMC9284622

The above article, published online on 12 October 2021, in Wiley Online Library (http://onlinelibrary.wiley.com/), has been retracted by agreement between the journal Editor in Chief, Professor Keith Harding; and John Wiley & Sons Ltd. Following an investigation by the publisher, all parties have concluded that this article was accepted solely on the basis of a compromised peer review process. In addition, further investigation by the publisher found that no consent statement from the patient depicted in Figure 3 had been included in the article and that the operational methods were not adequately described in the methodology. In view of the clear evidence of compromised peer review, the parties agreed that the paper must be retracted. The authors did not respond to our notice regarding the retraction.

